# Effects of *Claroideoglomus etunicatum* Fungi Inoculation on Arsenic Uptake by Maize and *Pteris vittata* L.

**DOI:** 10.3390/toxics10100574

**Published:** 2022-09-30

**Authors:** Guofei Pan, Yanyan Wei, Ningning Zhao, Minghua Gu, Bing He, Xueli Wang

**Affiliations:** Guangxi Key Laboratory for Agro-Environment and Agro-Products Safety, State Key Laboratory for Conservation and Utilization of Subtropical Agri–Bioresources, National Demonstration Center for Experimental Plant Science Education, College of Agriculture, Guangxi University, Nanning 530004, China

**Keywords:** AM fungi, arsenic, organic acid, maize, *Pterisvittata* L., phytoremediation

## Abstract

The intercropping of arsenic (As) hyperaccumulator Chinese brake fern (*Pterisvittata* L.) with maize (*Zea mays* L.) is being widely utilized to enhance phytoremediation without impeding agricultural production. Arbuscular mycorrhizal (AM) fungi can regulate the physiological and molecular responses of plants in tolerating heavy metal stress. We studied the effects of inoculation with AM fungi on As uptake by maize and *P. vittata* grown in soil contaminated with As. The results show that infection with the fungus *Claroideoglomus etunicatum* (Ce) increased the biomass of maize and *P. vittata*. Moreover, infection with Ce significantly reduced the accumulation of As and the coefficient for root–shoot transport of As in maize, whereas it enhanced the accumulation of As and coefficient for root–shoot transport of As in *P. vittata*. Infection with Ce led to a high content of available As in the soil planted with *P. vittata*, while there was a lower content of available As in the soil planted with maize. The different concentrations of available As in the soils suggest that inoculation with Ce may enhance the secretion of organic acids, particularly citric acid and tartaric acid, by maize roots and promote rhizosphere acidification, which then causes a decrease in As uptake by maize. Inoculation with Ce decreased the secretion of citric acid from *P. vittata* and promoted rhizosphere alkalization, which then caused an increase in As uptake by *P. vittata* and maize. Thus, co-combining AM fungi in the intercropping of the hyperaccumulator *P. vittata* with maize could be a promising approach to improving the efficiency of remediating As-contaminated soil.

## 1. Introduction

Heavy metal pollution in soil is a prominent environmental problem in China, particularly in east and southwest China [1,2]. Arsenic (As) is one of the primary heavy metal pollution elements in the soil. Nearly 2.7% of the farmland soil in China is polluted by As. Contamination of the soil with As will not only result in poisoned plants and seriously affect crop yields and quality but will also affect agricultural production and human health through the contamination of water sources [3]. As one of the three major maize (*Zea mays* L.) production areas in China, southwest China is an important maize production base. However, this part of China is a typical karst landform with many As ores and parent materials with a high content of As. There is a high background value of As in the soil, which poses a substantial challenge to safe agricultural production [4]. Currently, phytoremediation technology is the best method to reduce or eliminate the toxic effects of heavy metals in soil. It is widely used in the remediation of heavy metals in soil owing to its advantages of low cost and high income [5]. However, it is difficult to apply only a single phytoremediation technology for the effective remediation of moderately and lightly polluted soil. Because of the slow growth and low enrichment efficiency of hyperaccumulators, which are commonly used in phytoremediation, normal agricultural production is delayed, and the economic benefits of hyperaccumulator are low.

Some studies have found that the intercropping of a hyperaccumulator with crops can reduce the absorption of heavy metals in the crop, and the hyperaccumulator can be used in the phytoremediation of heavy metals without impeding agricultural production [6,7]. For example, Ma et al. [8] intercropped Chinese brake fern *(Pteris vittata* L.) with maize, which significantly reduced As absorption in maize and improved the rate of As removal by *P. vittata*. To further improve the remediation efficiency of *P. vittata*, inoculation with AM fungi was tested and found to significantly increase the biomass and As uptake by *P. vittata*, which in turn improved the efficiency of *P. vittata* in remediating As-contaminated soil [9,10,11,12]. Yang et al. [13] found that using AM fungi to inoculate dry rice (*Oryza sativa* L.) intercropped with black nightshade (*Solanum nigrum* Linn.) significantly reduced the cadmium content of dry rice while promoting cadmium uptake by *Solanum nigrum*. However, there are few reports on the effect of inoculating AM fungi on the intercropping system of *P. vittata* and maize.

Other studies have shown that organic compounds in root exudates can promote the As dissolution to improve the bioavailability of soil As its absorption by plants [14,15]. As described above, southwest China is part of a karst landform. In addition to the high background levels of As, clay minerals in the soil will also adsorb and accumulate a certain amount of As. In addition, the pH value of the soil dominated by limestone soil is relatively high, which results in the high availability of As. Therefore, reduction in the As availability in soil is one of the directions that can be used to enable agricultural production to be safely conducted in southwest China. The organic acids in root exudates can change the pH of rhizosphere soil and therefore the form of occurrence of As, which then affects its availability to organisms [16]. Organic acids contain carboxyl groups (-COOH) that can interact with metal ions and form chelates with metal ions through ion exchange and interaction to reduce the biological toxicity of heavy metals in soil [17]. Organic acid groups function as ligands of heavy metals. The absorption, transportation, and accumulation of heavy metals by plants are therefore linked with the presence of organic acids. However, there is no unified conclusion regarding the effect of organic acids on the absorption of heavy metals by plants. Moreover, the effect of AM fungi on the secretion of organic acids from and the accumulation of heavy metals in roots is unclear.

Therefore, the purpose of this study was to determine the effects of inoculating AM fungi on the As accumulation, organic acid secretion, and rhizosphere pH of a *P. vittata* and maize intercropping system. The results of this study will help in establishing the differences in organic acid metabolism between the hyperaccumulating plant (*P. vittata*) and the cash crop (maize) resulting from the influence of AM fungi and provide a theoretical basis to further understand the phytoremediation of As-contaminated soil by intercropping and AM fungal inoculation.

## 2. Materials and Methods

### 2.1. Plant Cultivation

The maize variety used in this experiment was Zhengda 999, *P. vittata*, an ecotype from Yunnan with a strong As enrichment capacity. Maize seeds were surface sterilized using 10% H_2_O_2_ for 10 min, thoroughly rinsed with deionized water, placed on a sheet of wet filter paper, germinated in the dark, and then cultured in pots. *P. vittata* was propagated by sprinkling its spores uniformly in a substrate that had been sterilized by autoclaving at 121 °C for 30 min. The substrate was kept moist enough during the culture period, and *P. vittata* seedlings about 10–15 cm tall with 4–5 leaves and showing robust and stable growth were selected for transplanting. Both corn and centipede grass were cultured with river sand that had been passed through a 20-mesh sieve and autoclaved at 121 °C for 30 min.

### 2.2. AMF Inoculants and Inoculation Methods

The AM strain tested was *Claroideoglomus etunicatum* (Ce), which was purchased from the Bank of Glomeromycota in China (BGC) of the Institute of Plant Nutrition and Resources, Beijing Academy of Agriculture and Forestry Sciences, and it had been collected from jujube gardens in Yantai city, Shandong province. In the early stage of the experiment, maize was used as the host, and the strain was expanded by sand culture in pots. After 4 months of propagation, the aboveground maize parts were removed. The roots were then cut into pieces, and the sand that contained fungal spores, hyphae, infected root segments, and other propagators was used as the inoculant.

### 2.3. Experimental Setup

#### 2.3.1. Effect of Ce Infection on the Secretion of Organic Acids from Maize/*P. vittate* Root

A sand culture pot experiment was used in the experiment, and the selected culture container was 22 cm × 29 cm (inner diameter × height) plastic bucket. A hole with a diameter of 28 mm was drilled near the edge of the bottom of the plastic barrel, and a rubber plug was placed inside this hole. A bent neck glass tube with an outer diameter of 1 cm and an angle of 120° was threaded into the rubber plug. One end of the glass tube in the barrel was wrapped with two layers of 300-mesh nylon gauze and fixed with a nylon rope. One end of the glass tube outside the barrel was connected to a latex tube with an inner diameter of 1 cm and a length of 10 cm, which served as a collection channel for the organic acids. The two types of plants used in this experiment were cultured separately. Four treatments were established: maize (Control); maize + Ce; *P. vittata* (Control); *P. vittata* + Ce. Each treatment had four replicates, which were grown for 45, 60, and 75 days. Each pot was filled with 11 kg of treated river sand, in which 300 g of AMF inoculants was sown layer by layer in the inoculation treatment, and the same amount of AMF inoculants was sown layer by layer in the Control treatment (sterilization under high temperature and damp heat at 121 °C for 2 h). After one week of growth, maize (“Control” and “maize + Ce”) was treated with 5 mg/L Na_3_AsO_4_·2H_2_O (aq) arsenic salts. *P. vittate* (“Control” and “*P. vittate* + Ce”) was treated with 30 mg/L Na_3_AsO_4_·2H_2_O (aq) arsenic salts. The plants were alternately irrigated with Na_3_AsO_4_·2H_2_O (aq) (pH 6.5) and complete Hoagland nutrient solution once every 3 days during the culture period. The plants were cultured in the glass greenhouse at Guangxi University (Nanning, China), and the light cycle followed that of natural sunlight. The other conditions of each treatment room were the same. The latex tube was clamped with a metal clamp during planting and then opened when the organic acids were to be collected.

#### 2.3.2. Effect of Ce Infection on the Rhizosphere pH of Maize and *P. vittata*

A sand culture pot experiment was used, and the two plants were cultured separately. Four treatments were set for the test, namely maize (Control), maize + Ce, *P. vittata* (Control) and *P. vittata* + Ce. Each treatment was replicated four times, and each basin was filled with 5 kg of river sand that had been autoclaved at 121 °C for 30 min. Among them, 150 g of bacterial agent was sown in layers in the inoculation treatment, and the same quality of sterilized bacterial agent was sown in layers in the Control treatment (sterilization for 2 h under high temperature and damp heat at 121 °C). After one week of growth, maize (“Control” and “maize + Ce”) was treated with 5 mg/L Na_3_AsO_4_·2H_2_O (aq) arsenic salts. *P. vittate* (“Control” and “*P. vittata* + Ce”) was treated with 30 mg/L Na_3_AsO_4_·2H_2_O (aq) arsenic salts. The plants were alternately irrigated with Na_3_AsO_4_·2H_2_O (aq) (pH 6.5) and complete Hoagland nutrient solution once every 3 days during the culture period. The plants were cultured in the glass greenhouse at Guangxi University, and the light cycle followed that of natural sunlight. The other conditions of each treatment room were the same.

The infection status of the maize and *P. vittata* roots were analyzed at 45 and 60 days after transplantation (refer to Section 2.4.1 for methods). The results showed that more than 30% of the maize and more than 25% of *P. vittata* were infected with mycorrhizae. These results indicate that a moderate level of infection of mycorrhizal maize and *P. vittata* root systems, i.e., that the host root systems were infected to a higher degree with Ce. After that, changes in the rhizosphere pH of maize and *P. vittata* at different time points (maize: 3 h, 6 h, 10 h, and 12 h; *P. vittate*: 2 h, 4 h, 6 h, 8 h, 10 h, and 12 h) were observed according to an in situ color development method (see Section 2.4.3 for details), and changes in the rhizosphere pH and proton secretion were quantitatively analyzed [18,19].

#### 2.3.3. Method of Collection of the Organic Acids

Three days before organic acid collection, the plants were incubated with 0.5 mM CaCl_2_ (aq) (pH 6.5) with the aim of washing away impurities and anions adsorbed on the sand. On the day before the organic acid collection, a cation exchange column (16 cm × 14 cm) was filled with 5 g H+ type cationic resin (Amberlite IR-120B; Organo Corp., Tokyo, Japan) and placed on an anion exchange column (16 nm × 14 cm) filled with 2 g of formic acid anion resin (Dowex 8100-200 mesh; Dow Chemical Company, Midland, MI, USA), washed with ultrapure water to remove bubbles, and stored at 4 °C. The organic acids were collected at 8:00. The metal clips were opened, and the plants were watered with 0.5 mM CaCl_2_ (aq) (pH 6.5). The outflow liquid was collected in the dark and then stored at 4 °C. The collected solution was successively passed through the cation anion resin, and the anion exchange resin was washed with ultrapure water to adsorb the anions. The anion exchange resin was then eluted three times with 5 mL of 2 M HCl. The eluent was evaporated at 40 °C in a rotary evaporator (n-1000v; Eyela Co., Inc., Tokyo, Japan). The concentrate was dissolved in 1 mL of ultrapure water, passed through a 0.45 µM water microporous filter membrane, and then measured by ion chromatography.

#### 2.3.4. Plant Sample Collection Method

The maize sample was divided into three parts, namely the roots, stems, and leaves, and two parts of *P. vittata* were harvested, namely the roots and shoots. All samples were successively washed with tap water and deionized water. The root system was immersed in PBS (1 mM K_2_HPO_4_, 5.0 mM MOPS, and 0.5 mM Ca[NO_3_]_2_; pH 6.0) precooled at 4 °C for 20 min to remove the As adsorbed on the root surfaces and then rinsed with deionized water. Approximately 0.5 g of fresh samples from various parts were frozen in liquid nitrogen, freeze dried under vacuum, and stored at −80 °C for characterization of the form of As. A small amount of the roots was also sampled and incubated in formaldehyde/alcohol/acetic acid (FAA) fixation solution to determine the colonization rate. The samples were weighed after the water on the sample surface was blotted dry with filter paper. The remaining samples were dried at 105 °C for 30 min, followed by drying at 65 °C to constant weight, and the dry weight of each plant part was then determined.

### 2.4. Sample Analysis

#### 2.4.1. Mycorrhizal Colonization

The plant roots were washed in FAA fixation solution, rinsed with deionized water, cut into segments of approximately 1 cm, and placed into 10 mL centrifuge tubes that had plugs.

The maize samples were incubated at 90 °C in a constant temperature water bath, rinsed with 10% KOH (aq) for 10 min, and washed three times with deionized water, which was then blotted off the samples. The root segments were acidified in 2% HCl (aq) for 5 min. The acid was removed, and the segments were blotted dry. A volume of 5% ink vinegar (blue black ink:vinegar = 1:19 [*v*/*v*]) was used to dye the samples in a 90 °C constant temperature water bath for 30 min. The dye was removed, and the samples were soaked in deionized water overnight.

Samples of *P. vittata* were incubated at 90 °C in a constant temperature water bath and rinsed with 20% KOH (aq) for 1 h. The alkali liquor was removed, and the samples were washed three times with deionized water. The samples were then blotted dry. A fresh solution of alkaline hydrogen peroxide was prepared by combining 567 mL of deionized water, 30 mL of 10% hydrogen peroxide, and 3 mL of ammonia. The samples were placed in this solution and incubated at room temperature for 45 min. They were then rinsed three times with deionized water and blotted dry. The acidification and dyeing steps were the same as those used on maize.

A total of 100 root segments were randomly selected for microscopic examination. The colonization rate calculation formula is as follows:

$$\mathrm{Colonization}\;\mathrm{rate}\left(\%\right)=\frac{\sum \left(0\%\times I0+10\%\times I1+20\%\times I2+\cdots \ldots 100\%\times I10\right)}{\mathrm{Total}\;\mathrm{number}\;\mathrm{of}\;\mathrm{root}\;\mathrm{segments}\;\mathrm{observed}}$$

where “I” is the number of root segments that were colonized, and “I0” is the number of root segments that were not colonized. “I1” is the number of root segments that were 1~10% colonized, and “I10” is the number of root segments that were 90% to 100% colonized.

#### 2.4.2. Determination of Organic Acid Content

Five organic acids, including malic acid, tartaric acid, phytic acid, oxalic acid, and citric acid, were analyzed by ion chromatography (ICS-5000; Dionex Corporation, Sunnyvale, CA, USA). A gradient elution method was used for separation and determination of these organic acids. The injection volume was 0.2 mL, and the flow rate was 1.2 mL·min^−1^. The concentration of each organic acid was calculated according to its corresponding peak area.

#### 2.4.3. Determination of pH and Proton Secretion in the Rhizosphere

Agar color indicator standard solutions were prepared in advance. The pH values of agar color indicator standards used for maize were 4.42, 4.65, 4.82, 5.00, 5.20, 5.41, 5.60, 5.83, 5.99, 6.20, 6.44, 6.64, 6.80, and 7.00. The pH values of agar color indicator standards used for *P. vittata* were 4.99, 5.23, 5.39, 5.62, 5.82, 6.01, 6.20, 6.40, 6.61, 6.79, 7.01, 7.20, 7.40, and 7.59. Image J software (NIH, Bethesda, MD, USA) was used for gray scale analysis, and a standard curve of the linear relationship between the pH value and the gray value of agar color indicator was prepared.

The maize root system was divided into three longitudinal rhizosphere areas during sampling for the root tip, middle, and base. Three to five points were taken from each area for the gray analysis, and the final pH value of rhizosphere was obtained by comparison with a standard curve. In combination with the method described by Rao et al. [18], the initial and final pH values of the agar color indicator were brought into the following formula, respectively, to determine the amount of protons secreted by the roots. A positive value represents rhizosphere acidification, and a negative value represents rhizosphere alkalization [19].

$$H^{+}=\frac{\left[\left(10^{-\mathrm{pHf}}-10^{-\mathrm{pHi}}\right)\right]\times 10^{9}\times P_{v}}{1000}$$

where “H^+^” is the amount of secreted protons (μmol); “pHi” is the initial pH value of the agar block; “pHf” is the final pH value of the agar block; “P_v_” is the volume of each pixel, and “P_v_” in this test is 0.01875 cm^3^.

#### 2.4.4. Content of As and P in the Plants

A total of 0.2 g of powdered dry plant sample was placed in a digestion tube, and 8 mL of concentrated HNO_3_ (GR) was added. The mixture was incubated overnight for predigestion. The samples were digested the next day using a microwave digestion instrument (MARS6; CEM Corporation, Matthews, NY, USA). The acid was removed using heat (120 °C, 120 min), and constant volume after cooling. There were two reagent blanks during this period, and the national standard substance gbw-07603 was used to control the quality of analysis of As in the plants. The treatment was the same as that of the sample. The content of As was determined by hydride generation atomic fluorescence spectrometry (HG-AFS, SA-20, Beijing Jitian Instruments Co., Beijing, China). P in plants was determined using an inductively coupled plasma emission spectrometer (ICP-5000, Beijing Spotlight Technology Co., Beijing, China). The recovery of As in the test was 97–105%.

#### 2.4.5. Determination of the Morphology of As in the Plant

Freeze-dried plant samples were ground to a uniform powder in liquid nitrogen. A total of 50 mg of the sample was weighed in a 10 mL centrifuge tube, and 5 mL of aqueous methanol (methanol: water =1:1 [*v*/*v*]) was added. The mixture was extracted by ultrasound treatment for 2 h in an ice bath. The ice bath was changed every 30 min to prevent the transformation of As, and the extract was shaken every 20 min. After 2 h, the extract was centrifuged at 4 °C at 13,000 rpm for 5 min and filtered, and the extraction process was repeated. After that, the residue was cleaned with 5 mL of deionized water, centrifuged, and filtered. The cleaning process was repeated twice. After the extraction solution had been mixed twice and the cleaning solution three times. The morphology was determined using high pressure liquid chromatography hydride generation atomic fluorescence spectrometry (HPLC-HG-AFS, E2695, WATERS Corporation, Milford, MA, USA).

### 2.5. Relevant Parameters

(1) Transfer coefficient (TF): (Heavy metal content in plant of shoot(mg/kg))/(Heavy metal content in plant root (mg/kg)).
(2) Effective transport coefficient (ETF): (Heavy metal content in plant of shoot (mg/kg) × Shoot biomass (g))/(Heavy metal content in plant root (mg/kg) × Root biomass (g)).
(3) Phosphorus arsenic ratio (P/As): (P content of plants (mg/kg)/(As content of plants (mg/kg)).
(4) As(III)/As(V): As(III) content in plant/As(V) content in plant.

### 2.6. Statistical Analysis

The test data were analyzed using Microsoft Excel 2010 (Redmond, WA, USA), and the data were tabulated using SPSS 21.0 (IBM, Inc., Armonk, NY, USA). Duncan test was used for multiple comparisons, and t-test was used for comparisons of two samples to test the significance of difference between treatments (*p* < 0.05).

## 3. Results

### 3.1. Effect of Inoculation with Ce on the Secretion of Organic Acids from Maize and P. vittata at Different Growth Stages

#### 3.1.1. Rates of Maize and *P. vittata* Mycorrhizal Colonization at Different Growth Stages

As shown in Table 1, the host root system was not infected by Ce at 45, 60, and 75 days when the plants were not inoculated. The rates of infection of maize and *P. vittata* roots inoculated with Ce ranged from 38.26~40.53% and 25.32~29.35%, respectively.

#### 3.1.2. Biomass of Maize and *P. vittata* at Different Growth Stages

Infection by Ce significantly affected the root and shoot dry weights of maize at different growth stages compared with the control (Table 2), with the exception of the maize root dry weight at 45 days. Infection with Ce typically increased the dry weight of maize roots and shoots by 1.20~1.64-fold and 1.20~1.26-fold, respectively. Similarly, infection with Ce also significantly increased the dry weight of *P. vittata* roots and shoots by 1.35~1.91-fold and 1.26~2.50-fold, respectively.

#### 3.1.3. The Content and Accumulation of As in Maize and *P. vittata* at Different Growth Stages

**Figure 1:**
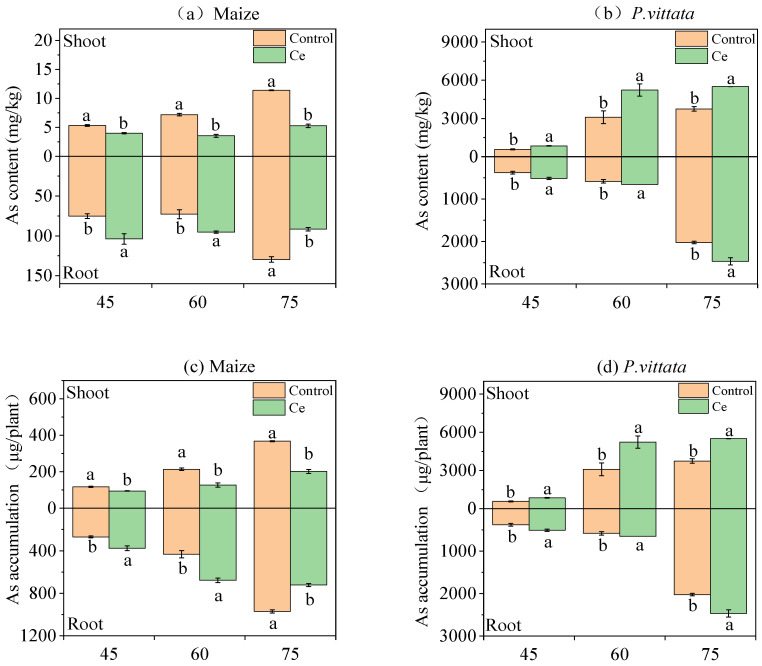
Effects of Ce colonization on As content (**a**,**b**)and accumulation (**c**,**d**) in maize and *P. vittata*, respectively, under As stress. As: arsenic; Ce, *Claroideoglomus etunicatum*. Lowercase letters “a” and “b” in the same group in the graph indicate significant differences between treatments (*p* < 0.05).

Ce infection of maize significantly increased the As content in roots and decreased the As content in shoots (Figure 1a). Compared with the control at 45 days, the As content in roots increased significantly by 1.38-fold, and the heavy arsenic content in shoots decreased significantly by 26.04%. Compared with the control at 60 days, the As content in roots increased significantly by 1.31-fold, and the As content in shoots decreased significantly by 50.72%. Compared with the control at 75 days, the As content in the roots and shoots decreased significantly by 29.31% and 53.69%, respectively. Similarly, Ce infection of maize significantly increased As accumulation in roots by 25.59~56.71% and decreased As accumulation in shoots by 19.26~45.19% at different growth stages (Figure 1c). In addition, As content and accumulation in maize roots after Ce inoculation were significantly higher than the control at 45 and 60 days, but significantly decreased at 75 days (Figure 1a,c).

Infection with Ce in *P. vittata* significantly increased the As content in roots and shoots at different growth stages (Figure 1b). Compared with the control, the root As content significantly increased by 14.67~341.09% and 48.21~443.73%, respectively. The highest As content in *P. vittata* roots (2574.45 mg/kg) and shoots (3556.92 mg/kg) was found 60 days after Ce infection. Similarly, the As accumulation in the roots and shoots of *P. vittata* infected by Ce increased significantly by 1.64~2.14-fold and 1.72~3.75-fold, respectively (Figure 1d).

#### 3.1.4. P/As of Maize and *P. vittata* at Different Growth Stages

**Figure 2:**
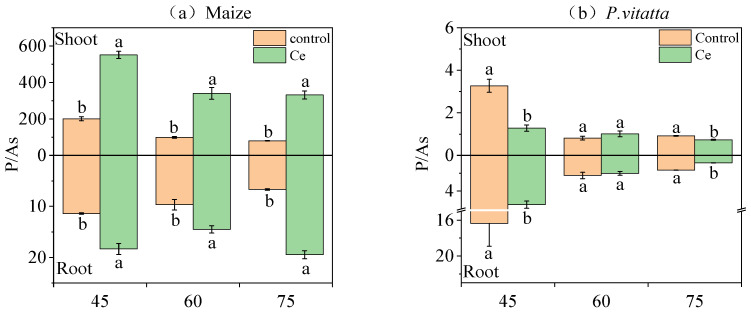
Effects of Ce colonization on the P/As of (**a**) maize and (**b**) *P. vittata* under arsenic stress. Ce, *Claroideoglomus etunicatum*; P/As, phosphorus content/arsenic content. Lowercase letters “a” and “b” in the same group in the graph indicate significant differences between treatments (*p* < 0.05).

The P/As of maize roots and shoots of maize infected with Ce at the three growth stages increased significantly by 1.50~2.91-fold and 2.75~4.15-fold, respectively, compared with the control (Figure 2a). The P/As in the *P. vittata* roots and shoots infected with Ce fungi decreased by 66.27% and 60.89%, respectively, at 45 days compared with the control (Figure 2b). At 60 days, Ce infection had no significant effect on the P/As in the *P. vittata* roots and shoots. The P/As in *P. vittata* roots and shoots decreased by 48.69% and 20.43% at 75 days, respectively.

#### 3.1.5. Effects of Ce Infection on the Content of As Species in Maize and *P. vittata* at Different Growth Stages

**Figure 3:**
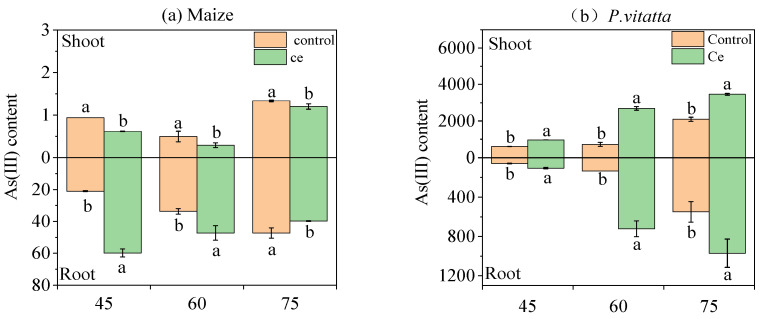

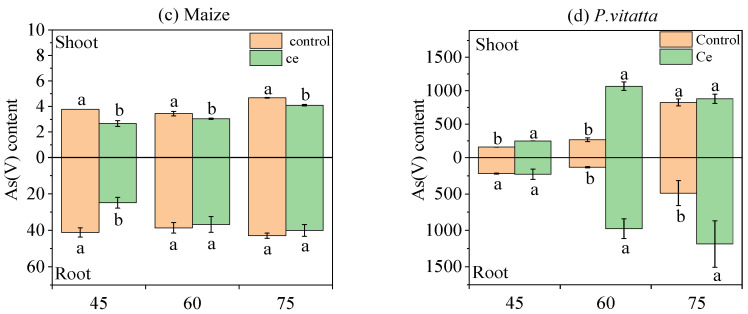
Effects of Ce colonization on the contents of As species in different parts of maize and *P. vittata* under As stress. As(III): (**a**,**b**); As(V): (**c**,**d**). As: arsenic; Ce, *Claroideoglomus etunicatum*. Lowercase letters “a” and “b” in the same group in the graph indicate significant differences between treatments (*p* < 0.05).

Inoculation with Ce significantly changed the distribution of As species in maize and *P. vittata* plants (Figure 3). After inoculation with Ce, the As(III) content in the maize roots increased by 2.84-fold and 1.40-fold at 45 and 60 days, respectively (Figure 3a). The As(V) content decreased by 39.56% and 4.88% at 45 and 60 days, respectively (Figure 3c). The distribution of As(III) in the aerial parts of maize decreased by 34.08% and 40.67% at 45 and 60 days, respectively, and the As(V) content decreased by 6.50% and 32.86%, respectively. In contrast, the As(III) content in the roots and shoot decreased by 15.73% and 9.98% at 75 days, respectively, and the As(V) content decreased by 6.76% and 12.42%, respectively. The As(III) contents in the roots and shoots of *P. vittata* increased significantly by 1.77~5.37-fold and 1.60~3.72-fold after inoculation with Ce, respectively (Figure 3b). In addition, the As(V) contents in the roots and shoots of *P. vittata* inoculated with Ce also increased significantly by 1.05~7.32-fold and 1.07~3.99-fold, respectively (Figure 3d).

Moreover, the ratio of As(III)/As(V) in the roots and shoots of maize and *P. vittata* was significantly affected by Ce infection (Table 3). It is apparent that, compared with the control, Ce infection at 45 days increased the ratio of As(III)/As(V) in the maize roots by 4.71-fold, and the ratio of As(III)/As(V) in the shoots decreased by 6.50% (Table 3). After 60 days of Ce infection, the ratio of As(III)/As(V) in the roots increased by 1.47-fold, and the ratio of As(III)/As(V) in the shoots decreased by 32.86%. After 75 days, the ratio of As(III)/As(V) in the maize roots decreased by 9.62%, and the ratio of As(III)/As(V) in the shoots increased by 2.79%. For *P. vittata*, the ratio of As(III)/As(V) in the roots increased by 70.37% at 45 days, the ratio of As(III)/As(V) in the roots decreased by 26.73% at 60 and 75 days, and the ratio of As(III)/As(V) in the shoots increased by 55.12% at 75 days.

#### 3.1.6. Translocation Factors of As in Maize and *P. vittata* at Different Growth Stages

As shown in Table 4, the As transport coefficient and the effective As transport coefficient of maize roots and shoots infected by Ce fungi decreased significantly by 42.86% and 41.86% at 45 days, respectively, compared with the control. The coefficient and effective coefficient for root–shoot transport of As in maize infected by Ce decreased significantly by 63.27% and 63.26% after 60 days, respectively. After 75 days, the coefficient and effective coefficient for root–shoot transport of As in maize infected by Ce decreased significantly by 34.57% and 26.29%, respectively. After 45 days, the coefficient and effective coefficient for root–shoot transport of As in *P. vittata* infected by Ce increased significantly by 20.86% and 92.96%, respectively. The coefficient and effective coefficient of for root–shoot transport of As in *P. vittata* increased significantly by 51.13% and 13.37%, respectively. After 75 days, the coefficient and the effective coefficient for root–shoot transport of As in *P. vittata* increased significantly by 20.54% and 47.85%, respectively.

#### 3.1.7. Effects of Ce Infection on the Secretion of Organic Acids from Maize and *P. vittata* Roots at Different Growth Stages

**Figure 4:**
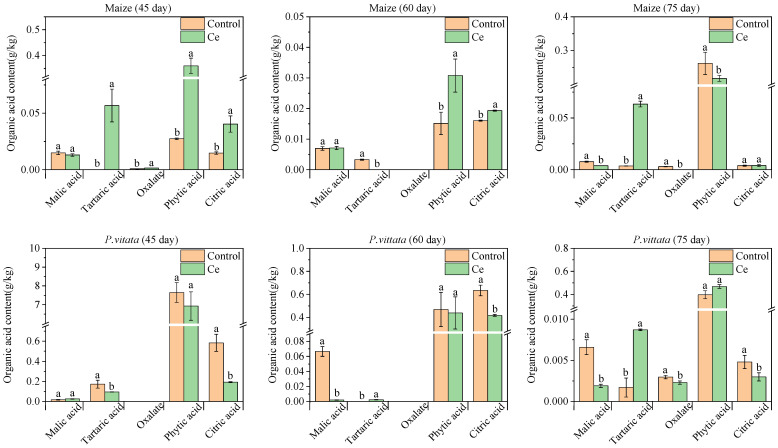
Effects of colonization by Ce on the types and content of organic acids secreted by maize and *P. vittata* under arsenic stress. Ce, *Claroideoglomus etunicatum*. Lowercase letters “a” and “b” in the same group in the graph indicate significant differences between treatments (*p* < 0.05).

The contents of malic acid, tartaric acid, oxalic acid, phytic acid, and citric acid secreted by the root system after the infection with Ce in maize and *P. vittata* were determined (Figure 4). Compared with the control, the contents of oxalic acid, phytic acid, and citric acid secreted by the roots of maize increased by 1.96-, 14.60- and 2.74-fold, respectively, 45 days after inoculation with Ce (Figure 4). However, there was no significant difference in the malic acid content. After 60 days, the contents of phytic acid and citric acid secreted by the maize roots increased significantly by 2.03- and 1.20-fold compared with the control, respectively, while the content of malic acid did not differ significantly (Figure 4). After 75 days, the contents of malic acid and tartaric acid secreted by maize roots increased significantly by 1.98- and 17.54-fold compared with the control, respectively, while the contents of phytic acid and citric acid did not differ significantly (Figure 4).

After 45 days, the infection of *P. vittata* by Ce significantly reduced the contents of tartaric acid and citric acid secreted by the roots by 44.75% and 66.89%, respectively, while there was no significant difference in the contents of malic acid and phytic acid (Figure 4). After 60 days, the contents of malic acid and citric acid secreted by the *P. vittata* roots infected by Ce decreased by 97.01% and 34.25% compared with the control, respectively, while the content of phytic acid did not differ significantly (Figure 4). The contents of malic acid, oxalic acid, and citric acid decreased by 71.09%, 23.27%, and 37.94% after 75 days, respectively, compared with the control, while the contents of tartaric acid increased by 4.99-fold, and there was no significant difference in the content of phytic acid (Figure 4).

### 3.2. Effects of Infection of Maize and P. vittata by Ce on the Rhizosphere pH

#### 3.2.1. In Situ Chromogenic Characteristics of Rhizosphere pH in Maize and *P. vittata*

**Figure 5:**
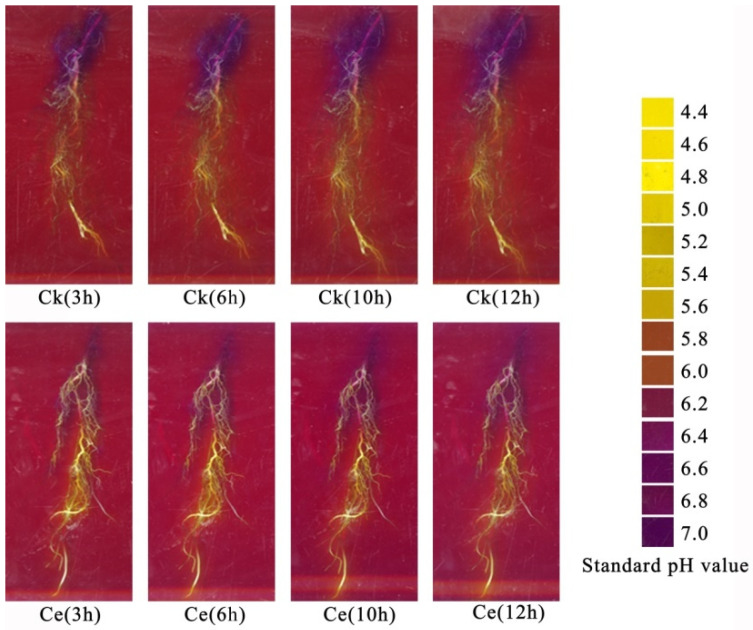
Changes in the rhizosphere pH of maize at different time points. Ce, *Claroideoglomus etunicatum*; Ck, control.

As shown in Figure 5, the standard pH diagram on the right after infection by Ce indicates that the maize root tips, middle roots, and base were more significantly acidified than the control at 3, 6, 10, and 12 h. As time progressed, the area of rhizosphere that was acidified gradually expanded, and the speed of acidification increased.

**Figure 6:**
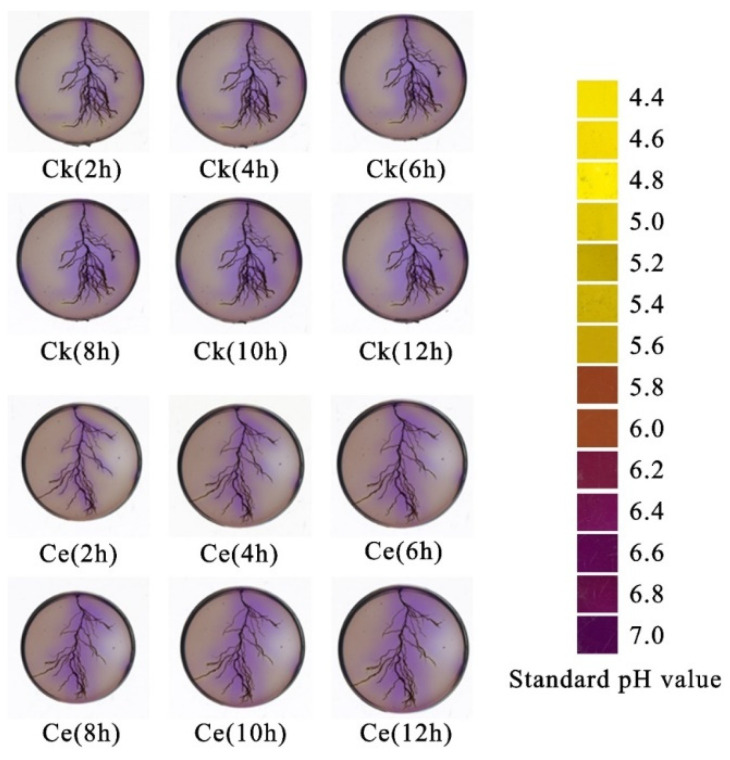
Changes in the rhizosphere pH of *P. vittata* at different time points. Ce, *Claroideoglomus etunicatum*; Ck, control.

Figure 6 shows the changes in the *P. vittata* rhizosphere pH. The standard pH diagram on the right indicates that infection by Ce significantly increased the degree of alkalization compared with the control at 2, 4, 6, 8, 10, and 12 h after infection. As time progressed, the area of rhizosphere that was alkalized gradually expanded, and the speed of alkalization increased.

#### 3.2.2. Quantitative Estimation of the Maize and *P. vittata* Rhizosphere pH

**Figure 7:**
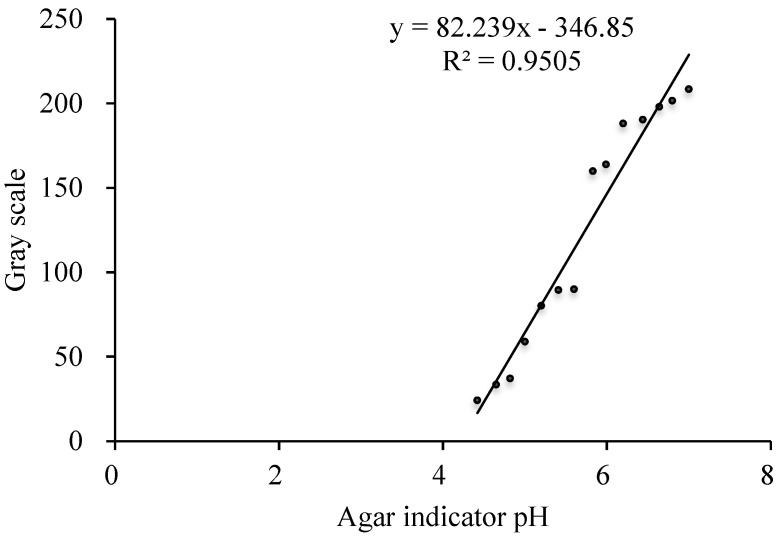
Calibration curve between the gray level and pH, linear calibration was obtained between pH 4.42 and 7.00 (maize).

An in situ color development test for determining the pH of the maize rhizosphere and the standard curve of the relationship between pH value and gray value of the agar color indicator is shown in Figure 7. The secretion of H^+^ by the roots is shown in Table 5. Compared with the control, the pH values of the root tips, middle roots, and base decreased by 0.24, 0.14, and 0.04 units, respectively. The secretion of protons from the root tip and middle root increased by 89.02% and 47.63%, respectively, and the secretion of OH^−^ by the base decreased by 4.52% at 3 h. The pH value of the maize root tips, middle roots, and base all decreased by 0.17 and 0.18 units at 0 and 6 h, respectively. The secretion of protons from the root tips and middle at 0.03 units increased by 58.22% and 72.98%, respectively, and the secretion of OH^−^ by the base decreased by 2.54%. At 10 h, the pH values of the root tips, middle roots, and base decreased by 0.11, 0.36 and 0.03 units, respectively, while the proton secretion from the root tips and middle roots increased by 36.37% and 100.69%, respectively, and the secretion of OH^−^ by the base decreased by 2.75%. At 12 h, the pH values of the root tips, middle roots, and base decreased by 0.16, 0.37, and 0.08 units, respectively. The secretion of protons from the root tips and middle roots increased by 62.30% and 104.64%, respectively, and the secretion of OH^−^ by the base decreased by 4.98%.

**Figure 8:**
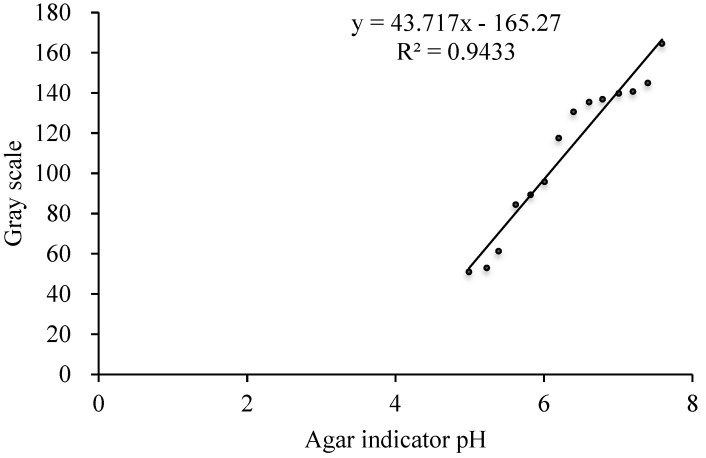
Calibration curve between the gray level of color and pH in *P. vittata*.

In the in situ color development test for determining the pH of the *P. vittata* rhizosphere, the standard curve of the relationship between pH value and gray value of agar color indicator is shown in Figure 8, and the secretion of H^+^ by the roots is shown in Table 6. At 2, 4, 6, 8, 10, and 12 h after inoculation with Ce, the *P. vittata* rhizosphere pH decreased by 0.12, 0.22, 0.24, 0.24, 0.39, and 0.29 units, respectively, and the secretion of OH- by the rhizosphere increased by 4.28%, 5.81%, 5.37%, 5.45%, 7.80%, and 4.79%, respectively, compared with the control.

## 4. Discussion

### 4.1. Effects of Ce on the Secretion of Organic Acids from Maize and P. vittata

AM fungi can directly or indirectly improve the physiological metabolism and growth of plants under heavy metal stress, regulate the microecological environment in the plant rhizosphere, and affect the tolerance of plants to heavy metals [20,21]. The results show that the biomass of maize and *P. vittata* increased significantly after inoculation with Ce under As stress. The mycorrhizal infection rate of the two plants increased as the growth cycle extended, indicating that the dependence of plants on mycorrhizae gradually increases with the extension of the growth cycle under As stress. Some studies have shown that the increase in root exudates after inoculation with AM fungi could be related to the increase in biomass. The increase in biomass led to an increase in the amount of photosynthetic products, and some photosynthetic products were transformed into root exudates [22]. Our results do not confirm this, which could be due to the differences in plant types [23].

Stress can change the secretion of organic acids, such as malic acid and oxalic acid, from the *P. vittata* root system, which increases the pH of rhizosphere soil [24]. The species and quantity of amino acids and organic acids in the plant root exudates, as well as rhizosphere pH, will change the bioavailability of heavy metals, consequently affecting the absorption and accumulation of heavy metals by plants [25,26]. For example, Chen et al. [27] found that organic acids secreted by moso bamboo (*Phyllostachys edulis* [Carriere] J. Houzeau) roots promote the adsorption of Pb and Cd while inhibiting the adsorption of Cu and Zn in soil. Wang et al. [28] showed that the accumulation of organic acids stimulates the transfer of Cd, Zn, and Cu from soil to vegetables but inhibits the absorption of Pb by vegetables. Our results show that Ce promotes the secretion of organic acids in maize roots and reduces the accumulation of As in maize under As stress. In contrast, it decreases the secretion of organic acids in the roots of *P. vittata* and increases the accumulation of As in *P. vittata*. It has been reported that the organic acids secreted by plant roots can reduce the rhizosphere soil pH by 0.2~0.5 units [29]. The soil pH value is the primary factor that affects the availability of As. Low pH values can inhibit As availability in soil that is highly polluted with As [30]. To further verify whether inoculation with Ce affects the secretion of organic acids from root and the rhizosphere pH, we studied the effect of Ce inoculation on rhizosphere pH. The results show that the pH value of maize root tips decreased by 0.25 units after inoculation with Ce (Table 5). In contrast, the pH value of *P. vittata* roots increased by 0.29 units (Table 6). Therefore, inoculating maize with Ce can promote the acidification of its rhizosphere, which reduces the availability of As. Thus, this reduces the absorption of As by maize. Inoculating *P. vittata* with Ce can increase the alkalinity of its rhizosphere, which improves the availability of As in the rhizosphere, thereby improving the absorption of As by *P. vittata*. However, the primary organic acids affecting the rhizosphere pH have not been identified. Wang et al. [31] found that oxalic acid and malic acid are the primary organic acids that are secreted and cause changes in the pH of the Masson’s pine (*Pinusmassoniana* Lamb.) rhizosphere. Huang et al. [32] found that there is a significant linear negative correlation between the organic acid content and rhizosphere pH and that malic acid, citric acid, and the total organic acid contents are all strongly correlated with pH (greater than −0.98, *p* < 0.01). In addition, it was reported that malic acid, oxalic acid, and citrate, secreted by plants, affect the plant As absorption by chelating heavy metals, while the tricarboxylic acid anion citric acid more strongly chelates heavy metals [33,34,35]. Our results show that inoculation with Ce increased citric acid secretion from maize in three growth stages (2.74-, 1.2-, and 1.05-fold, respectively) under As stress reduced citric acid secretion from *P. vittata* at the corresponding stages (66.89%, 34.25%, and 37.94%, respectively). Therefore, citric acid could be the primary organic acid that affects the maize rhizosphere pH. Moreover, citric acid can chelate As in the rhizosphere and reduce its availability.

In addition to affecting the rhizosphere pH, the organic acids secreted by plant roots, including phytic acid and oxalic acid, can also dissolve mineral As in the soil; phytic acid can more effectively migrate than oxalic acid at the same concentration [36,37,38,39,40]. For example, Liang et al. [41] found that the As content in the root system of *P. vittata* increased by 12.0% after the exogenous addition of oxalic acid. In our study, the content of phytic acid in the maize roots increased during the early stage (45 and 60 days) after inoculation with Ce, but the secretion of phytic acid was inhibited in the late stage (75 days). This is consistent with its trend of accumulating in maize. Moreover, after inoculation with Ce, the secretion of phytic acid by *P. vittata* decreased during the early stage (45 and 60 days) (no significant difference) but began to increase in the late stage (75 days). However, the low oxalic acid content in this study could be due to differences in culture substrate and plant age [23]. The production of phytate as a root exudate is considered to be a special property of ferns and plays an important role in improving the absorption of arsenic and nutrients by plants [38,42]. Phytic acid secreted by *P. vittata* roots can promote the complexation of phosphorus and As, the upregulation of a phosphorus transporter, and the absorption of As by *P. vittata* [43,44]. Therefore, we hypothesized that phytic acid could be an organic acid that controls the absorption of As by plants. Inoculation with Ce inhibits the secretion of phytic acid by maize, thereby reducing its absorption of As, while it does not affect the secretion of phytic acid from *P. vittata* but inhibits the secretion of other organic acids, promotes rhizosphere alkalization, and increases the availability of As.

In this study, there was a significant increase in the content of tartaric acid secreted by the roots of both plants after inoculation with AM fungi. After 75 days, the tartaric acid content of maize increased by 17.54-fold compared with that of uninoculated maize, and the tartaric acid content of *P. vittata* increased by 4.99-fold. Moreover, tartaric acid was only detected during the early stage (45 and 60 days) of maize infected by Ce, while tartaric acid was also detected during the late stage (75 days) of maize without infection. This suggests that there could be a delay in the secretion of tartaric acid from maize roots not infected by Ce, and infection by Ce promotes the synthesis and secretion of organic acids and thus eliminates this delay. Currently, there are relatively few reports on the role of tartaric acid in detoxification. Huang et al. [32] showed that tartaric acid and citric acid are the primary organic acids secreted by castor (*Ricinus communis* L.) roots under copper stress, and they could be a mechanism for plants to detoxify Cu. Tao et al. [45] concluded that tartaric acid is a unique root exudate of *Sedum alfredii* H. and enhances the migration and absorption of cadmium in this plant. Moreover, the secretion of tartaric acid is positively correlated with Cd accumulation.

### 4.2. Effects of Ce on As Uptake of by Maize and P. vittata

The results show that the As content in different parts of maize and *P. vittata* varied in different growth stages after inoculation with Ce. The arsenic content in maize roots increased significantly at 45 and 60 days and decreased significantly at 75 days, while the content of As in the shoots decreased significantly at three growth stages. However, the As content in the *P. vittata* roots and shoots increased significantly in the three growth stages after inoculation with Ce. Many studies have found that inoculation with AMF in an intercropping system can increase the absorption of heavy metals by hyperaccumulating plants and either reduce or at least suppress increases in the absorption of heavy metals by cash crops (nonhyperaccumulating plants) when the soil is heavily polluted by metals [46,47]. This could be caused by the change in root microenvironment and the competition of available heavy metals in plants. In addition, phosphorus and As are homologous elements, and their absorption is somewhat competitive. The absorption of phosphorus can reduce the absorption and accumulation of As to some extent. Xu et al. [48] found that AM fungi enhance the absorption of P by *Medicago truncatula* and decreased the concentration of As in the buds and roots, which resulted in the increased dry weight of buds and roots. In this study, the P/As values of all the parts of maize significantly increased during the three growth stages after inoculation with Ce. In contrast, the P/As values of all the parts of *P. vittata* decreased significantly during the three growth stages.

### 4.3. Effect of Ce Inoculation on the Accumulation of Arsenic in Maize and P. vittata

It has been reported that AM fungi can regulate the activity of arsenate reductase in plant roots and then affect the form of arsenic present in roots, but this is not the key factor that determines the difference in As accumulation between mycorrhizal and nonmycorrhizal maize [46]. Our results in Table 3 show that Ce promotes maize roots in reducing As(V) to As(III). However, the accumulation of total As in the maize with mycorrhizae was significantly higher than that in nonmycorrhizal maize during the early and middle stages (45 and 60 days). Ce could have promoted the reduction of As(V) to As(III) in maize roots, causing it to be partitioned and thus improving the arsenic tolerance of maize roots [49,50]. Typically, As(III) is more phytotoxic than As(V), but the detoxification mechanism of most plants involves a reduction of As(V) to As(III) followed by its partitioning into vacuoles [51]. Yu et al. [46] found that mycorrhizal fungi accumulate more As(V) than nonmycorrhizal fungi after AMF inoculation in maize. In this study, this phenomenon only appeared in the later stage (75 days). The reason could be that maize itself is a plant that is rich in As(V), and inoculation with Ce may have slowed down the process of As(V) enrichment [52,53].

As the growth period of *P. vittata* progressed, the proportion of mycorrhizal and nonmycorrhizal plants that accumulated As(III) increased, but the proportion of mycorrhizal As(III) was always lower than that of the nonmycorrhizal plants (Table 3). The analysis of the characteristics of accumulation of total As in *P. vittata* roots (Table 2) suggest that the reason could be that the mycorrhizal plants reduce As(V) more slowly than they absorb As(V), or that Ce promotes the transport of As(III) from the mycorrhizae to the aerial parts of the plant. It could be that both of these processes occur simultaneously. Previous studies have shown that *P. vittata* primarily absorbs As(V), accumulates As(V) in the root system, and partially reduces it to As(III), which is then transported to the shoots. The inoculation of AM fungi can improve the absorption and enrichment of arsenic in *P. vittata* and improve the activity of arsenate reductase [12,54,55,56].

Marin et al. [57] showed that the form of As not only determines the plant utilization of As in rice but also its migration or translocation in plants. In our study, the inoculation of Ce significantly inhibited As transport from the maize roots to the shoots, while promoting the transport of As from the *P. vittata* roots to the shoots (Table 4). As shown in Figure 3, As(III) primarily accumulates in maize roots, while As(V) primarily accumulates in the shoots. After inoculation with Ce, As(III) in the roots increased, while the accumulation of As in the shoots was reduced. In contrast, *P. vittata* primarily accumulates As(V) in the roots and As(III) in the shoots. Inoculation with Ce significantly increased the accumulation of total As and As(III) in the shoots. Therefore, we hypothesize that Ce promotes the reduction of As(V) into As(III) in the maize and *P. vittata* roots, but As(III) in the maize roots can be directly fixed in the roots, while As(III) in *P. vittata* roots is more easily transferred to the aboveground plant parts [58,59]. Trotta et al. [60] hypothesized that the higher translocation factor in mycorrhizal plants could be caused by the higher transport of As to the thallus and/or mechanisms of As exclusion in roots. Our research results support the former hypothesis.

## 5. Conclusions

The results of our study showed that inoculation with Ce reduced the accumulation of As in maize and promoted its absorption by *P. vittata*. The reason for this difference could be that Ce promotes the secretion of organic acids, such as citric acid and phytic acid, by maize roots, causing them to become acidified. This results in a reduction in the concentration of available As in the rhizosphere. In contrast, the inoculation of *P. vittata* with Ce reduced the secretion of organic acids, such as citric acid, in the roots. This results in their alkalization and increases the concentration of available As in the rhizosphere. Alternatively, Ce could reduce the coefficient and effective coefficient of root to shoot transport As in maize, which would inhibit the ability of maize to transport As. However, increasing the coefficient and effective coefficient of As transport from the roots to shoots of *P. vittata* improved the transport of As in this plant.

In summary, these results provide a good basis for the remediation of soil contaminated with As by intercropping *P. vittata* with crops. However, the two crops were grown in monoculture in this study. Thus, the potential benefits of intercropping the two crops were not fully realized. Therefore, field trials are merited to further test the feasibility of this model.

## Figures and Tables

**Table 1:** Effects of colonization by Ce on the mycorrhizal colonization rate of maize and *P. vittat* under arsenic stress in different periods.

	Treatments		Colonization%
Maize	45 days	Control	0.00 ± 0.00 b
		Ce	38.26 ± 0.34 a
	60 days	Control	0.00 ± 0.00 b
		Ce	39.01 ± 0.32 a
	75 days	Control	0.00 ± 0.00 b
		Ce	40.53 ± 1.38 a
*P. vittata*	45 days	Control	0.00 ± 0.00 b
		Ce	25.32 ± 1.69 a
	60 days	Control	0.00 ± 0.00 b
		Ce	27.32 ± 0.69 a
	75 days	Control	0.00 ± 0.00 b
		Ce	29.35 ± 1.63 a

Note: Different letters for the same plant indicate significant differences between the control and Ce inoculation at the same stage (*p* < 0.05). Ce, *Claroideoglomus etunicatum*. Lowercase letters “a” and “b” in the same group in the graph indicate significant differences between treatments (*p* < 0.05). The same below.

**Table 2:** Effects of colonization by Ce on the maize and *P. vittata* biomass under arsenic stress in different periods.

	Treatments		Dry Weight (g)	
			Root	Shoot
Maize	45 days	Control	3.60 ± 0.08 a	21.85 ± 0.03 b
		Ce	3.64 ± 0.05 a	23.47 ± 0.37 a
	60 days	Control	5.95 ± 0.07 b	29.64 ± 0.48 b
		Ce	7.12 ± 0.17 a	35.58 ± 1.07 a
	75 days	Control	7.51 ± 0.11 b	32.20 ± 0.26 b
		Ce	8.89 ± 0.03 a	38.10 ± 0.28 a
*P. vittata*	45 days	Control	1.07 ± 0.05 b	1.47 ± 0.05 b
		Ce	1.49 ± 0.04 a	3.68 ± 0.36 a
	60 days	Control	5.70 ± 0.33 b	15.22 ± 0.95 b
		Ce	10.89 ± 0.73 a	19.15 ± 0.99 a
	75 days	Control	8.37 ± 0.16 b	16.83 ± 0.38 b
		Ce	11.30 ± 0.36 a	27.79 ± 1.42 a

Note: Different letters in same list of the same plant indicate significant difference (*p* < 0.05). Ce, *Claroideoglomus etunicatum*.

**Table 3:** The ratios of As(III)/As(V) in different parts of maize after colonization by Ce under As stress in different periods.

	Treatments		Parts	As(III)/As(V)
Maize	45 days	Control	Root	0.51
			Shoot	0.25
		Ce	Root	2.40
			Shoot	0.23
	60 days	Control	Root	0.87
			Shoot	0.14
		Ce	Root	1.28
			Shoot	0.10
	75 days	Control	Root	1.10
			Shoot	0.28
		Ce	Root	0.99
			Shoot	0.29
*P. vittata*	45 days	Control	Root	0.27
			Shoot	3.87
		Ce	Root	0.46
			Shoot	3.88
	60 days	Control	Root	1.01
			Shoot	2.71
		Ce	Root	0.74
			Shoot	2.53
	75 days	Control	Root	1.13
			Shoot	2.54
		Ce	Root	0.82
			Shoot	3.94

Note: As, arsenic; Ce, *Claroideoglomus etunicatum*.

**Table 4:** Effects of Ce colonization on the characteristics of arsenic accumulation in maize and *P. vittata* under arsenic stress in different periods.

Treatments			Translocation Factor (TF)	Effective Translocation Factor (ETF)
			Root–Shoot	Root–Shoot
Maize	45 days	Control	0.07 ± 0.004 a	0.43 ± 0.03 a
		Ce	0.04 ± 0.001 b	0.25 ± 0.02 b
	60 days	Control	0.10 ± 0.010 a	0.51 ± 0.07 a
		Ce	0.04 ± 0.002 b	0.19 ± 0.02 b
	75 days	Control	0.09 ± 0.002 a	0.38 ± 0.01 a
		Ce	0.06 ± 0.003 b	0.28 ± 0.02 b
*P. vittata*	45 days	Control	1.39 ± 0.08 b	2.13 ± 0.27 b
		Ce	1.68 ± 0.03 a	4.11 ± 0.25 a
	60 days	Control	5.30 ± 0.53 b	13.61 ± 0.02 b
		Ce	8.01 ± 0.57 a	15.43 ± 0.49 a
	75 days	Control	1.85 ± 0.08 b	3.72 ± 0.07 b
		Ce	2.23 ± 0.05 a	5.50 ± 0.33 a

Note: Different letters in the same list of the same plant indicate significant differences (*p* < 0.05). Ce, *Claroideoglomus etunicatum*.

**Table 5:** Effects of colonization by Ce on the rhizosphere pH and amount of protons secreted by maize under arsenic stress.

Treatments	Parts	Time (h)	Initial pH	pH Value	Proton Secretion (nmol/h)
Control	Root tip	3	6.00	5.25	28.71
		6	6.00	5.26	14.04
		10	6.00	5.34	6.67
		12	6.00	5.42	4.34
	Middle part	3	6.00	5.39	19.49
		6	6.00	5.47	7.38
		10	6.00	5.59	2.94
		12	6.00	5.63	2.08
	Basal	3	6.00	6.52	−4.36
		6	6.00	6.57	−2.28
		10	6.00	6.61	−1.42
		12	6.00	6.71	−1.26
Ce	Root tip	3	6.00	5.01	54.27
		6	6.00	5.09	22.22
		10	6.00	5.23	9.10
		12	6.00	5.26	7.04
	Middle part	3	6.00	5.25	28.78
		6	6.00	5.29	12.77
		10	6.00	5.38	5.90
		12	6.00	5.43	4.26
	Basal	3	6.00	6.48	−4.17
		6	6.00	6.54	−2.22
		10	6.00	6.58	−1.38
		12	6.00	6.63	−1.20

Note: A positive value in the proton secretion column indicates that H+ is secreted, and a negative value indicates that OH^−^ is secreted. Ce, *Claroideoglomus etunicatum*.

**Table 6:** Effects of Ce colonization on the pH and amount of protons secreted in the *P. vittata* rhizosphere.

Treatment	Time (h)	Initial pH	pH Value	Proton Secretion (nmol/h)
Control	2	6.00	6.83	−7.98
	4	6.00	6.89	−4.09
	6	6.00	6.92	−2.75
	8	6.00	6.94	−2.08
	10	6.00	6.93	−1.66
	12	6.00	7.06	−1.43
Ce	2	6.00	6.95	−8.33
	4	6.00	7.11	−4.33
	6	6.00	7.14	−2.90
	8	6.00	7.18	−2.19
	10	6.00	7.32	−1.79
	12	6.00	7.35	−1.49

Note: A positive value for proton secretion indicates the secretion of H^+^, while a negative value indicates the secretion of OH^−^. Ce, *Claroideoglomus etunicatum*.

## Data Availability

The data presented in this study are available within the article.
